# Resource Optimization in the Examination of Macroscopic Normal Colon Mucosa

**DOI:** 10.1055/a-2926-4399

**Published:** 2026-08-11

**Authors:** Kamilla M.B. Johannesen, Eva Gravesen, Thomas Blixt, Wojciech Cebula, Lars K. Munck, Anne-Marie K. Fiehn

**Affiliations:** 1 Department of Pathology 53140 Zealand University Hospital RoskildeRoskildeDenmark; 2 Department of Clinical Medicine 4321 University of CopenhagenCopenhagenDenmark; 3 Department of Pathology 53176 Herlev HospitalHerlevCapital Region of DenmarkDenmark; 4 Department of Surgery 524788 Zealand University Hospital KogeKøgeRegion ZealandDenmark; 5 Department of Internal Medicine 91909 Zealand University Hospital Nykøbing FNykøbing FalsterRegion ZealandDenmark; 6 Department of Gastroenterology 524788 Zealand University Hospital KogeKøgeRegion ZealandDenmark

**Keywords:** endoscopy, histology, histopathology, inflammation, microscopic colitis

## Abstract

**Background and Study Aims** Colonoscopy with biopsies is an essential procedure in the diagnostic workup of patients with chronic diarrhea. If the intestinal mucosa appears macroscopically normal or near-normal, tissue samples are retrieved from right and left segments of the colon and sometimes also from the ileum. Biopsies are sent to histologic analysis in individual containers. The aim of this retrospective study was to examine whether the current procedure could be simplified by merging biopsies in one container without affecting the diagnostic quality.

**Patients/Materials and Methods** Biopsies from patients with an endoscopically normal colon mucosa were included; 250 with a study diagnosis of microscopic colitis (MC) and 233 patients with a study diagnosis of non-MC. H&E-stained microscopy slides from the right and left colon were evaluated by two pathologists, and histologic concordance between the right and left colon was determined.

**Results** In 232 (92.8%) patients with MC and 229 (98.3%) patients with non-MC, the diagnosis was identical in the right and left colon. In six (2.4%) patients from the MC group, biopsies from both segments were within the MC spectrum, while in 12 (4.5%) patients, the histologic characteristics for MC were only fulfilled in one segment of the colon. Biopsies from the ileum were without clinical relevance.

**Conclusions** We suggest merging biopsies from the right and left colon of patients with macroscopically normal mucosa into a single container and refraining from obtaining ileal biopsies when MC is suspected, reducing resource consumption in the endoscopy unit and the pathology department. The results support a transition toward a more sustainable diagnostic procedure.

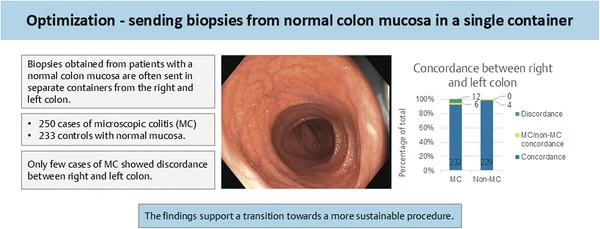

## 1 Introduction

Every day, a high number of colonoscopies are performed, and biopsies are obtained from a macroscopically normal or near-normal mucosa. The indication is frequently chronic, watery, nonbloody diarrhea, which is a common symptom with many etiologies. The single-most important reason for taking biopsies from patients with chronic, watery diarrhea and a macroscopically normal or near-normal mucosa with only subtle changes, including minor cat-scratch lesions, mucosal scarring, nodularity, mild edema, or erythema, is to either confirm or exclude microscopic colitis (MC), as the histologic pattern is very characteristic. 1 2 In around 10% of the patients, a diagnosis of MC is established. 3 4 5 6 Histologic examination of biopsies from the remaining patients rarely reveal the cause of the symptoms. 3 5 6 7 Other indications for colonoscopy include constipation, changes in bowel habits, anemia, or other vague symptoms 8 9 in which the colonic mucosa is often found to be macroscopically normal, and histopathologic analysis is of limited value.

Until recently, international guidelines recommended retrieving two biopsies from each segment of the colon (coecum/ascending, transverse, descending, and sigmoid colon), corresponding to a minimum of eight biopsies, from patients with clinical suspicion of MC, reflecting the fact that the mucosa appears normal or near-normal and that the microscopic changes were assumed to be patchy. 10 11 12 Lately, it has been shown that the changes are present in almost all biopsies from all segments of the colon. Accordingly, it is now generally accepted that only two biopsies from the right and left segments of the colon, respectively, are sufficient to establish a diagnosis of MC. 13 14 15 This was also recommended in the latest guidelines published by the European Microscopic Colitis Group (EMCG), which further specifies that the biopsies should be sent in separate containers. 16 This relies on the assumption that the number of immune cells is higher in the right segment of the colon, and therefore the pathologist needs to know the exact anatomic origin of the biopsies. 12 17 However, the histopathologic diagnostic criteria for MC do not differ between the right and left segments. Thus, the histopathologic evaluation and a final diagnosis of MC are independent of the localization of the changes in the colon, and the practice of sending the biopsies separately might be redundant.

**Fig. 1:**
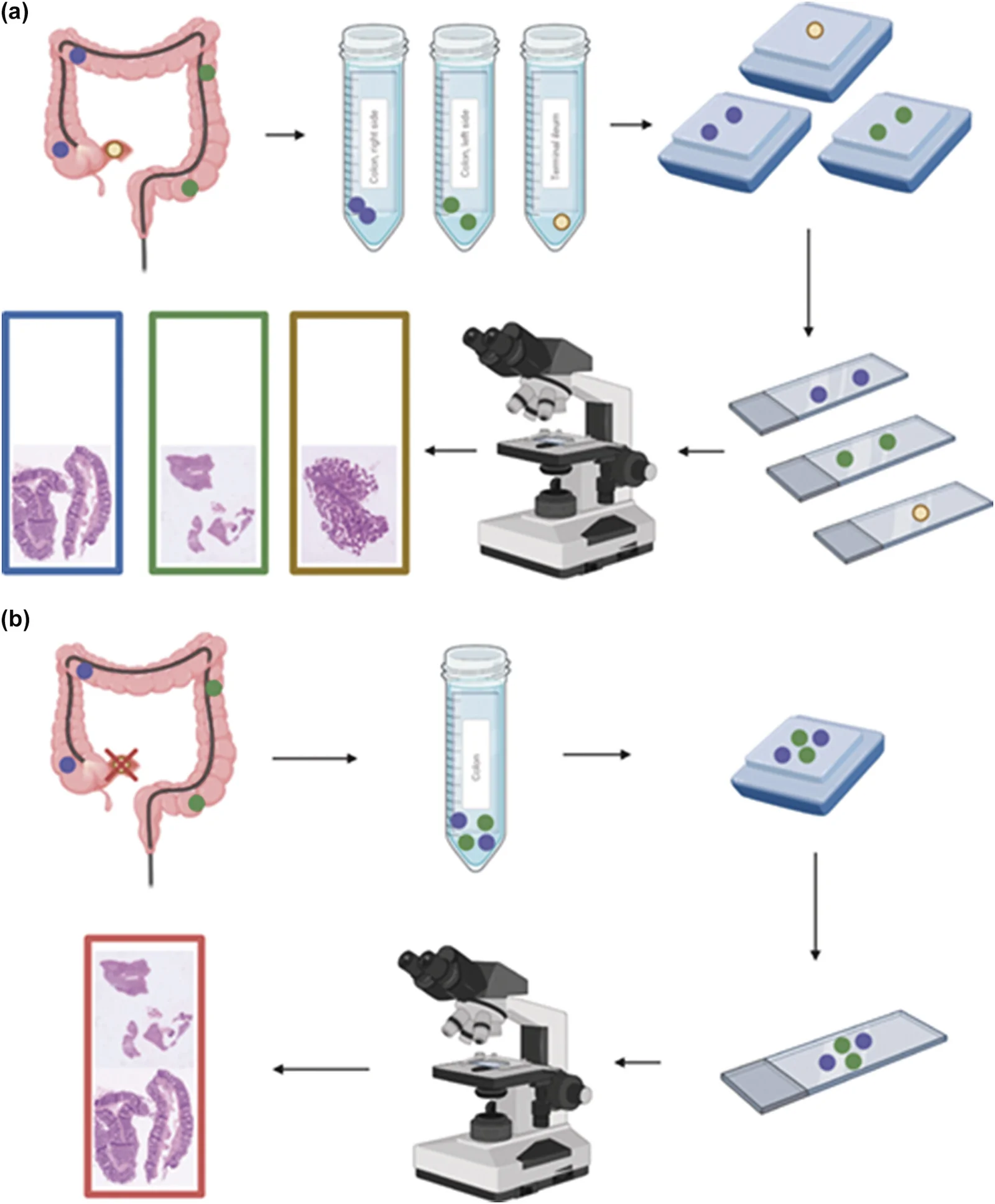
( **a** ) Currently, the procedure for patients with chronic watery diarrhea is to take biopsies from the right and left segments of the colon and submit them in separate containers. An ileal biopsy is often obtained. The tissue will be embedded in three paraffin blocks (one from each segment and one from the ileum) at the Department of Pathology, where sections will be cut and stained from each block and analyzed individually by the pathologist. Source: Created in BioRender. Kanstrup-Fiehn, A. (2026) https://BioRender.com/rb2vtrt . ( **b** ) The study investigates the possibility of taking the same number of biopsies from the colon but embedding them all in a single block and skipping the ileal biopsy altogether. Source: Created in BioRender. Kanstrup-Fiehn, A. (2026) https://BioRender.com/8al9vxz .

The primary aim of this study was to determine whether the current procedure with random biopsies from a macroscopically normal colon mucosa being sent in separate containers from the right and left segments of the colon could be changed to merging the same number of biopsies into a single container. Secondarily, it was assessed whether biopsies from the terminal ileum provided essential diagnostic information. The current and the proposed alternative workflows are illustrated in Fig. 1 .

## 2 Materials and Methods

### 2.1 Selection of Patients

This retrospective study was planned to include colon biopsies from 500 consecutive patients examined in Region Zealand, Denmark, from January 1, 2022 onward. The patients were divided into 250 individuals with biopsies that had been signed out with a diagnosis of MC, including microscopic colitis incomplete (MCi), and a control group of a comparable size consisting of patients without an MC diagnosis. The control group was included to assess whether a change in the current procedure with the merging of the random biopsies from the two segments of the colon mucosa could imply a risk of missing any significant diagnoses among non-MC patients.

Patients in the MC group were identified in the pathology registry by SNOMED codes for lymphocytic colitis (LC) or collagenous colitis (CC), while the control group consisted of patients with biopsies obtained from right and left colon mucosa. The pathology requisition was studied for clinical information, and only patients with a macroscopic normal or near-normal colon mucosa, and with biopsies from at least the right and left segments of the colon submitted separately, were included. Many patients had simultaneous polypectomies performed, and the polypectomy specimens were not included in the study. Biopsies from more than two locations were also allowed. Biopsies from the right segment were defined as being proximal to the left flexure. Patients <18 years, and patients with a previous histologic diagnosis compatible with chronic inflammatory bowel disease (IBD) in the pathology registry were not included.

Following inclusion, the National Database of Non-Consent to the Use of Tissue Samples for Scientific Purposes was checked, and patients who had prohibited the use of tissue were excluded. Next, patient charts were checked, and patients with a clinical diagnosis of IBD, although absent histopathologic findings, were also excluded. Finally, cases with very low quality of the microscopy slides were also excluded.

### 2.2 Histological Evaluation

Hematoxylin and Eosin (H&E)-stained microscopy slides were retrieved from the archive, pseudonymized, and assessed independently by two gastrointestinal (GI) pathologists (one from Region Zealand and one from the Capital Region), blinded to clinical information, including colon segment. In cases with multiple sections from the same paraffin block, a resident pathologist selected one representative section. Biopsies from colonic mucosa were categorized as either normal mucosa/nonspecific changes, chronic inflammation, acute inflammation, LC, incomplete lymphocytic colitis (LCi), CC, incomplete collagenous colitis (CCi), neoplasia/cancer, or others. Biopsies from ileal mucosa were categorized as either normal mucosa/nonspecific changes, chronic inflammation, acute inflammation, microscopic colitis/incomplete microscopic colitis, or others. The histopathologic characteristics for the categories can be seen in **Table S1** .

One diagnosis was registered for each biopsy location by each of the two pathologists. In case of disagreement, a consensus diagnosis was established. Next, one final diagnosis was determined for each patient. In cases where the diagnosis differed between the two segments of the colon, the final study diagnosis was made based on a hierarchy with CC > LC > CCi > LCi > acute inflammation > chronic inflammation > normal/nonspecific changes. For patients with biopsies from more than two locations, one diagnosis for the right segment and one diagnosis for the left segment were established.

Age, sex, and number of biopsies from each location were extracted from the pathology requisition. It was registered whether the colonoscopy had been performed in private practice or a hospital unit. For patients with examination at a hospital unit, additional clinical symptoms and endoscopic findings were obtained from the patient charts.

### 2.3 Data Presentation and Statistics

Data are presented separately for patients with a final study diagnosis of MC and non-MC. For baseline characteristics, the categorical data are presented with frequencies and percentages and evaluated for statistical significance using a Fisher’s exact test. Continuous variables are presented with medians and interquartile ranges (IQR). For all calculations, *p* -values < 0.05 were considered significant. Concordance of histologic diagnosis between the right and left colon is presented according to the final study diagnosis (MC and non-MC). All statistical analyses and data visualization were performed with Excel, version 2507, and R version 4.5.1.

### 2.4 Ethics

The study was approved by the Local Committee on Health Research Ethics (SJ-1048) and registered in the Region Zealand record of processing activities related to scientific research projects (REG-2024-16304) according to the Declaration of Helsinki and Danish law.

## 3 Results

### 3.1 Basic Characteristics and Categorization of Included Patients

In all, 500 patients with an original signed-out diagnosis of either MC (250 cases) or non-MC (250 cases) were included. Of these, 17 patients were excluded due to insufficient quality of the slides ( *n* = 3), a prior diagnosis of IBD ( *n* = 11), age younger than 18 ( *n* = 1), or registration in the electronic health record or the National Database of Non-Consent to the Use of Tissue Samples for Scientific Purposes ( *n* = 2). The study cohort consisted of 250 patients with a final diagnosis of MC and 233 patients with a non-MC diagnosis. The two pathologists agreed on the diagnosis in 809 of 1002 biopsies (80.7%) from the colon and 286 of 352 biopsies (81.3%) from the ileum, leaving 193 and 66 slides for consensus. The original signed-out diagnosis of the cases was used as a third observer to establish a final diagnosis based on the majority, but for 32 slides from the colon, and zero biopsies from the ileum, a complete reevaluation and discussion between the two pathologists were needed, as the original diagnosis and the evaluation by the two study pathologists were divided in three different diagnostic categories. The 32 colon slides originated from 22 patients.

The median age was 72 years in the MC group and 58 years in the non-MC group. In all, 169 (67.6%) were women in the MC group, and 140 (60.1%) were women in the non-MC group. The median number of biopsies taken in both groups was 8 (Q1; 6 and Q3; 9), ranging from 4 to 17 and 2 to 15 in the MC and non-MC groups, respectively. The number of biopsy locations in the colon was likewise comparable between the two groups, with a large majority of patients having only two biopsy locations in both groups ( Table 1 ). Totally, 82 (17.0%) of the cases originated from private practice, while 401 (83.0%) of the patients had the procedure performed at a hospital unit.

**Table 1:** Clinicopathologic characteristics of the patients according to the final study diagnosis (MC and non-MC), colon.

	MC ( *N* = 250)	Non-MC ( *N* = 233)
**Age in years; median (Q1;Q3)**	72 (62;78)	58 (42;70)
** Sex, *n* (%) **		
Male	81 (32.4%)	93 (39.9%)
Female	169 (67.6%)	140 (60.1%)
**Number of biopsies, median (Q1;Q3)**	8 (6;9)	8 (6;9)
**Number of biopsy locations, n (%)**		
Two	229 (91.6%)	225 (96.6%)
Three	18 (7.2%)	7 (3.0%)
Four	0 (0.0%)	1 (0.4%)
Five	3 (1.2%)	0 (0.0%)
** Concordance between right and left segments of the colon, *n* (%) **		
Complete concordance	232 (92.8%)	229 (98.3%)
Concordance, MC/non-MC	6 (2.4%)	4 (1.7%)
Discordance	12 (4.8%)	–
**Histopathologic findings**		
Normal/nonspecific, *n* (%)	–	219 (94.0%)
Chronic inflammation, *n* (%)	–	11 (4.7%)
Acute inflammation, *n* (%)	–	3 (1.3%)
LC, *n* (%)	114 (45.6%)	–
LCi, *n* (%)	11 (4.4%)	–
CC, *n* (%)	115 (46%)	–
CCi, *n* (%)	10 (4%)	–
Secondary finding of neoplasia, *n* (%)	3 (1.2%)	4 (1.7%)

### 3.2 Clinicohistologic Analysis of Cases

#### 3.2.1 Histologic Diagnosis

In the MC group, 114 (45.6%) and 11 (4.4%) patients were categorized with a final diagnosis of LC or LCi, while 115 (46%) and 10 (4%) had a final diagnosis of CC or CCi, respectively. In the non-MC group, 219 (94.0%) were categorized with a final diagnosis of normal or nonspecific reactive changes, 11 (4.7%) with chronic inflammation, and 3 (1.3%) with acute inflammation ( Table 1 ). For three patients in the MC group and four patients in the non-MC group, an unexpected finding of a serrated polyp ( *n* = 4) or an adenoma with low-grade neoplasia ( *n* = 3) was recorded. Four of the seven patients with incidental findings of serrated polyps or adenomas had a simultaneous polypectomy performed, increasing the risk of cross-contamination between specimens.

#### 3.2.2 Concordance between Histologic Diagnosis in the Right and Left Segments of the Colon

Complete concordance in the histologic diagnosis between the right and left colon was seen in 232 (92.8%) patients in the MC category and in 229 (98.3%) patients in the non-MC category. When reducing the number of diagnostic categories to two, MC and non-MC, respectively, the concordance increased to 238 (95.2%) cases in the MC category. The corresponding percentage in the non-MC category obviously increased to 100%, as a histologic MC diagnosis in either segment would mean the case was categorized as MC and therefore no longer part of the non-MC group.

The clinicopathologic characteristics of patients with different diagnoses in the left and right segments of the colon, but where both locations showed either MC ( *n* = 6) or non-MC ( *n* = 4) in both segments (MC/non-MC concordance) are shown in Table 2a . In the MC group, four patients had nonbloody, watery diarrhea, and one had reported a change in stool habits with a duration of more than 4 weeks. Table 2b shows the histologic diagnosis of the patients with MC/non-MC discordance between the right and left segments. All 12 patients were categorized with a final histologic diagnosis of MC ( *n* = 4) or MCi ( *n* = 8). Only two patients had reported nonbloody, watery diarrhea, and three a change in stool habits with a duration exceeding four weeks. Additionally, 10 of the patients in the group had been discussed and re-evaluated by the two study pathologists due to disagreement in either one or both segments of the colon, suggesting that the histologic evaluation had been challenging.

**Table 2a:** The histopathologic diagnosis according to each segment of the colon with the established final diagnosis in bold. All cases represent discordance between the two sides of the colon although both sides were classified as either non-MC or MC/MCi.

Patient no.	Right side	Left side	Indication for colonoscopy	Macroscopic finding(s)
*Non-MC subgroup*				
**1**	Normal/nonspecific	**Chronic inflammation**	Nonbloody diarrhea, changes in stool habits with a duration > 4 weeks	Normal
**2**	**Chronic inflammation**	Normal/nonspecific	Changes in stool habits with a duration >4 weeks, weight loss	Polyps
**3**	**Chronic inflammation**	Normal/nonspecific	Nonbloody diarrhea, weight loss, radiologic findings	Normal
**4**	**Chronic inflammation**	Normal/nonspecific	Pain	Normal
*MC subgroup*				
**5**	LC	**CC**	Nonbloody diarrhea, changing consistency, weight loss	Normal
**6**	**LC**	LCi	Blood in stools/rectal bleeding, anemia	Polyps
**7**	LCi	**CCi**	Changing consistency, changes in stool habits with a duration > 4 weeks	Hemorrhoids
**8**	CCi	**CC**	Nonbloody diarrhea, constipation, pain	Diverticula
**9**	**CCi**	LCi	Nonbloody diarrhea, changes in stool habits with a duration >4 weeks	Diverticula
**10**	**CC**	CCi	Nonbloody diarrhea, changes in stool habits with a duration >4 weeks	Hemorrhoids

**Table 2b:** The histopathologic diagnosis according to each segment of the colon with the established final diagnosis in bold. All cases represent discordance between the two sides of the colon, where one side was classified as MC/MCi, and the other as non-MC.

Patient no.	Right side	Left side	Indication for colonoscopy	Macroscopic finding(s)
**1**	**LCi**	Normal/nonspecific	Screening/follow up	Diverticula
**2**	**CC**	Normal/nonspecific	Rectal bleeding/blood in stools, screening/follow up	Polyps
**3**	**CCi**	Normal/nonspecific	Screening/follow up	Diverticula
**4**	**LCi**	Normal/nonspecific	Changes in stool habits with a duration >4 weeks	Polyps
**5**	**CCi**	Normal/nonspecific	Changes in stool habits with a duration >4 weeks	Diverticula, hemorrhoids, wall thickening
**6**	**CCi**	Normal/nonspecific	Nonbloody diarrhea	Normal
**7**	**CCi**	Normal/nonspecific	Radiologic findings	Normal
**8**	Normal/nonspecific	**CC**	Radiologic findings	Edema, polyps
**9**	**CCi**	Normal/nonspecific	Nonbloody diarrhea, changes in stool habits with a duration >4 weeks	Polyps
**10**	Normal/nonspecific	**CCi**	Weight loss	Diverticula, polyps
**11**	**CCi**	Normal/nonspecific	Changes in stool habits with a duration >4 weeks	Normal
**12**	**CC**	Chronic inflammation	Screening/follow up	Erythema/redness/irritation

### 3.3 Clinical Symptoms and Endoscopy Findings

The indications for colonoscopy in the cohort were diverse ( Table 3 ). The most common indication in both groups was nonbloody diarrhea, stated in 68.6% and 40.3% of patients in the MC and non-MC groups, respectively ( *p* < 0.001). The second-most common indication was a change in stool habits with a duration exceeding four weeks. This indication was mentioned in 50.9% and 30.9% of the patients in the MC and non-MC groups, respectively ( *p* < 0.001). Furthermore, significantly more patients in the MC group had experienced weight loss; 16.4% in the MC group and 8.3% in the non-MC group ( *p* = 0.016). In the non-MC group, there was a statistically significant higher frequency of constipation, changed consistency of stools, pain, nonspecific abdominal discomfort, rectal bleeding/blood in stools, and anemia.

**Table 3:** Clinical indications and macroscopic findings from patients examined at a hospital unit.

	MC ( *N* = 220)	Non-MC ( *N* = 181)	No. of patients ( *n* = 401)	*p* -Value	OR (95% Confidence Interval)
**Indications**					
** Nonbloody diarrhea, *n* (%) **	151 (68.6%)	73 (40.3%)	224 (55.9%)	**<0.001**	3.23 (2.10 - 4.99)
** Constipation, *n* (%) **	1 (0.5%)	7 (3.9%)	8 (2.0%)	**0.035**	0.11 (0.00 - 0.90)
** Changing consistency, *n* (%) **	18 (8.2%)	33 (18.2%)	51 (12.7%)	**0.004**	0.40 (0.20 - 0.76)
** Changes in stool habits with a duration >4 weeks, *n* (%) **	112 (50.9%)	56 (30.9%)	168 (41.9%)	**<0.001**	2.31 (1.50 – 3.57)
** Pain, *n* (%) **	26 (11.8%)	54 (29.8%)	80 (20.0%)	**<0.001**	0.32 (0.18 – 0.54)
** Nonspecific abdominal discomfort, *n* (%) **	13 (5.9%)	31 (17.1%)	44 (11.0%)	**<0.001**	0.30 (0.14 – 0.62)
** Mucus in stools, *n* (%) **	8 (3.6%)	8 (4.4%)	16 (4.0%)	0.799	0.82 (0.26 – 2.55)
** Bloody diarrhea, *n* (%) **	3 (1.4%)	2 (1.1%)	5 (1.2%)	1	1.24 (0.14 – 14.95)
** Rectal bleeding/blood in stools, *n* (%) **	8 (3.6%)	24 (13.3%)	32 (8.0%)	**<0.001**	0.25 (0.09 – 0.59)
** Suspicion of IBD, *n* (%) **	1 (0.5%)	2 (1.1%)	3 (0.75%)	0.591	0.41 (0.01 – 7.93)
** Anemia, *n* (%) **	4 (1.8%)	11 (6.1%)	15 (3.7%)	**0.033**	0.29 (0.07 – 0.99)
** Weight loss, *n* (%) **	36 (16.4%)	15 (8.3%)	51 (12.7%)	**0.016**	2.16 (1.11 – 4.41)
** Screening/follow up, *n* (%) **	17 (7.7%)	5 (2.8%)	22 (5.5%)	**0.045**	2.94 (1.01 – 10.41)
** Radiologic findings, *n* (%) **	10 (4.5%)	4 (2.2%)	14 (3.5%)	0.277	2.10 (0.59 – 9.35)
** Other, *n* (%) **	0 (0.0%)	2 (1.1%)	2 (0.5%)	0.203	0 (0-4.38)
**Macroscopic findings**					
** Normal, *n* (%) **	96 (43.6%)	95 (52.5%)	191 (47.6%)	**0.088**	0.70 (0.46 – 1.06)
** Near-normal/inflammation NOS, *n* (%) **	1 (0.5%)	1 (0.6%)	2 (0.5%)	1	0.82 (0.01 – 64.84)
** Erythema/redness/irritation, *n* (%) **	22 (10.0%)	11 (6.08%)	33 (8.3%)	0.201	1.71 (0.77 – 4.04)
** Edema, *n* (%) **	21 (9.5%)	5 (2.8%)	26 (6.5%)	**0.007**	3.70 (1.32 – 12.84)
** Blurring of vascular pattern, *n* (%) **	12 (5.5%)	3 (1.7%)	15 (3.7%)	0.063	3.41 (0.90 – 19.15)
** Friability, *n* (%) **	6 (2.7%)	0 (0.0%)	6 (1.5%)	**0.034**	Inf (0.98 – Inf)
** Ulcerations/erosions/fibrinous plaques, *n* (%) **	7 (3.2%)	3 (1.7%)	10 (2.5%)	0.522	1.95 (0.44 – 11.84)
** Diverticula, *n* (%) **	59 (26.8%)	48 (26.5%)	107 (26.7%)	1	1.02 (0.64 – 1.63)
** Polyp(s), *n* (%) **	52 (23.6%)	46 (25.4%)	98 (24.4%)	0.727	0.91 (0.56 – 1.47)
** Hemorrhoids, *n* (%) **	19 (8.6%)	10 (5.5%)	29 (7.2%)	0.251	1.61 (0.69 – 4.00)
** Wall thickening, *n* (%) **	4 (1.8%)	3 (1.7%)	7 (1.7%)	1	1.10 (0.18 – 7.60)
** Angiodysplasia, *n* (%) **	1 (0.5%)	3 (1.7%)	4 (1.0%)	0.332	0.27 (0.01 – 3.42)
** Other, *n* (%) **	2 (0.9%)	1 (0.6%)	3 (0.7%)	1	1.65 (0.09 – 97.91)

*Note: The percentages exceed 100% in both groups, as some patients presented with more than one indication or macroscopic finding.*

Table 3 also shows the macroscopic findings in the two groups. A completely normal colonic mucosa was the most frequently reported feature in both groups, with a prevalence of 43.6% in the MC group and 52.5% in the non-MC group. Minor changes such as erythema/redness/irritation, edema, blurring of the vascular pattern, and friability were more common in the MC group than in the non-MC group.

### 3.4 Ileal Biopsies

Ileal biopsy was obtained in 47.7% of patients with a diagnosis of MC and 52.3% of patients with a study diagnosis of non-MC. The finally established diagnoses of the ileal biopsies are shown in Table 4 . Most patients were classified as normal/nonspecific changes; 83.3% in the MC group and 97.3% in the non-MC group. Biopsies from the terminal ileum were suspicious for MC in 13 (7.7%) of the MC cases and in none of the non-MC cases.

**Table 4:** Clinicopathologic characteristics of the patients according to the final diagnosis (MC and non-MC), ileum.

	MC ( *n* = 168)	Non-MC ( *n* = 184)
**Age in years; median (Q1;Q3)**	69 (58;77)	55 (39.75;66)
** Sex, *n* (%) **		
Male	52 (31.0%)	78 (42.4%)
Female	116 (69.0%)	106 (57.6%)
** Normal/nonspecific, *n* (%) **	140 (83.3%)	179 (97.3%)
** Chronic inflammation, *n* (%) **	13 (7.7%)	4 (2.2%)
** Acute inflammation, *n* (%) **	2 (1.2%)	1 (0.5%)
**MC/MCi**	13 (7.7%)	0 (0.0%)

## 4 Discussion

Colonoscopy with biopsies from patients with a variety of symptoms, including chronic watery diarrhea, is a very common procedure that causes a massive workload and associated use of resources in the healthcare system. We hypothesized that the diagnostic quality would not be reduced, and the final diagnosis and following treatment of patients with macroscopic normal mucosa would remain unchanged, whether the biopsies are sent in two separate containers as currently recommended or merged in one single container.

The concordance between the histopathologic diagnoses in biopsies from the right and left segments from a macroscopically normal or near-normal colon mucosa was determined in a cohort of 483 retrospective and consecutively included patients, consisting of 250 patients with a histologically determined study diagnosis of MC and 233 patients with a histologically determined study diagnosis of non-MC. Concordant histologic diagnoses between the two segments of the colon were seen in 93% of the patients in the MC group and 98% of the non-MC patients. If only discriminating between a final diagnosis of MC or non-MC, concordance increased to 95% and 100%, respectively. That different subtypes of MC between the two segments were seen in a few cases is not surprising, as this has also been described in previous European studies. 18 19 In the clinical setting, discrimination between subtypes is not critical, as the treatment is independent of subtype. 16 Previous studies report similar high concordance rates between biopsies from the right and left segments in patients with MC, as we have found in this study. 20 21 However, these studies did not include a control group of non-MC patients, which is essential when considering a change in the procedure when obtaining biopsies from a normal mucosa, where you do not know the histological diagnosis in advance of microscopy.

For the complete cohort, the MC/non-MC discordance rate in this study was 2.5% between the two segments ( *n* = 12). In four and eight patients, the histologic criteria for an MC or MCi diagnosis, respectively, were only fulfilled in biopsies from one segment of the colon, while biopsies from the other segment were categorized as normal/nonspecific reactive changes or chronic inflammation. A histologic diagnosis of MC and especially MCi requires careful correlation to the clinical presentation. Several differential diagnoses should always be considered, which was not possible in this study, as the two pathologists were blinded to clinical information, and thus, some cases might have been classified differently in a routine setting. Merging biopsies could imply a risk of missing a few cases of MC/MCi, as the changes would probably appear more discrete when combined with an equal amount of normal tissue, but on the other hand, this might reduce the number of cases that would otherwise be wrongly classified as MC/MCi by the pathologist. Only 2 of the 12 patients with a divergent diagnosis between the two sides were registered as suffering from nonbloody diarrhea, and a further 3 with a final study diagnosis of MCi had a reported change in stool habits, which might include diarrhea. Thus, a maximum of five patients had MC and would benefit from treatment, while the remaining seven patients did not have any clinical symptoms pointing toward a diagnosis of MC. This finding could address a tendency to overdiagnosis due to the lack of clinical correlation. Furthermore, in the clinical routine, the pathologist often has access to clinical information, reducing the risk of missing a diagnosis of MC even if the changes are only present in half of the biopsies. As around 45% of MCi patients experience remission on placebo treatment and also tend to experience relapses slightly more rarely than patients with MC, this might signal an overall milder disease course or reflect more uncertainty of the histologic diagnosis. 22 23

Under normal conditions, the right side of the colon mucosa harbors a higher number of inflammatory cells compared with the left side. 12 17 A thin basement membrane with a thickness of up to 3 μm is located beneath the surface epithelium throughout the colon. 24 25 Knowing the origin of a biopsy might be beneficial if subepithelial fibrosis is present as several differential diagnoses to CC are more common in the sigmoid colon and rectum including mucosa prolapse, radiation damage, and ischemic colitis. 1 14 However, these disorders are usually accompanied by more pronounced macroscopic changes. The diagnostic criteria for MC and MCi are the same irrespective of the location in the colon and should therefore not influence the diagnosis. Having said this, knowledge of the location might be useful in few challenging borderline cases but is probably particularly used by pathologists to differentiate normal colon mucosa from mucosa with nonspecific reactive changes that have little clinical relevance.

In both the MC and non-MC groups, a few patients were diagnosed with serrated polyps or adenomas with low-grade neoplasia through random biopsies. Merging biopsies from the two segments would eliminate the possibility of knowing whether the location was the right or left segment of the colon. However, a precise location would not be possible either by preserving the biopsies in two materials.

Biopsies from macroscopically normal-appearing ileal mucosa were of very limited value. In 91% of the cases, the histology was normal or near normal. A minority of patients with MC also had changes in the ileum that were suspicious for MC or acute or chronic inflammation. In no case did the histologic evaluation contribute with crucial information. This is in concordance with previous reports. 26 27 28 One study evaluated ileal biopsies, including almost 7,500 patients with normal macroscopic mucosa. Active ileitis, intraepithelial lymphocytosis, chronic active ileitis, erosions, ulcers, or granulomas were only rarely reported. 29

Chronic watery diarrhea is a very common symptom affecting up to 5% of the adult population at any given time, and was also the most commonly reported symptom in this cohort. 30 Biopsies from the patients are mandatory to establish a diagnosis of MC. 1 2 Existing guidelines recommend taking biopsies from both the right and left segments of the colon and sending them as separate materials to the Department of Pathology. 11 16 Only around 10% of the patients with biopsies from a macroscopically normal colon mucosa will be diagnosed with MC, meaning that biopsies from the majority will not provide an explanation of the symptoms. 3 5 6 7 As MC has a severely negative impact on the quality of life, 31 and most of the patients respond well to treatment with budesonide, it is crucial to identify the patients. 16 32

A limitation of this study is the retrospective design. The biopsies were already mounted on two glass slides, and thus, it was not possible to exactly mimic the circumstances we wanted to investigate, where all biopsies were mounted on one glass slide and in this way the present study only represents an indirect evaluation of the suggested procedural change. Furthermore, the two pathologists had only one HE-stained section available from each paraffin block, which might explain some of the discrepancies between the original diagnosis made in clinical routine and the final diagnosis. Another limitation is the lack of clinical information upon review, as correlating the clinical symptoms to the histopathologic findings is crucial in making an MC diagnosis. Additionally, around 17% of the cohort was examined in private practice with no access to patient charts.

Within several areas of medicine, there is a tendency to build on more diagnostic tests while the removal of analyses is rarer. Combined with an aging population needing health care, expenses will continue to rise if new procedures are continually added.

A recent study by Leung et al. estimated that almost 10 min of working time would be saved at the pathology department by reducing the number of specimen containers by one. On top of this, time would be saved by the endoscopist. 33 The time saved is probably even higher in university departments with a teaching obligation. Furthermore, material use would be reduced. Costs are calculated differently worldwide but as this is a large patient group, the savings would be considerable.

The primary aim of this study was to evaluate whether the current procedure can be changed from sending separate biopsies from each segment of the colon to instead merging them in only one container, as this could simplify the procedure and reduce resource consumption. The suggested change of procedure in this study seems to retain the current level of quality while lessening the overall workload and time spent diagnosing this patient group, freeing up time for both the endoscopy unit and the pathologist, with minimal or probably no risk of missing a histologic diagnosis. It is important to remember that if focal changes are present (other than the nonspecific changes characteristic of MC), biopsies from separate locations should not be merged.

In conclusion, we suggest that biopsies from the right and left segments of macroscopic normal colon mucosa can be merged and examined as a single material, and biopsies from macroscopic normal ileal mucosa can be omitted. This can minimize the resources spent evaluating biopsies from this patient group significantly, including both material and time consumption. Thus, the results support a transition toward a more sustainable diagnostic procedure with a reduced consumption of resources. We recommend the results be validated in a prospective cohort.

## Supplementary Material

Ergänzendes Material Supplementary Material
