# Supplementary material for: Resource Optimization in the Examination of Macroscopic Normal Colon Mucosa
Source: Endosc Int Open. 2026 Aug 11;14:a29264399. doi: 10.1055/a-2926-4399 (PMC13494745; doi:10.1055/a-2926-4399)
Supplement: Supplementary file 1 [file 10-1055-a-2926-4399_29267591.pdf]

Supplementary Material: Resource optimization in the examination of macroscopic normal colon mucosa

Authors: Kamilla Maria Bech Johannesen MD, Eva Gravesen MD, PhD, Thomas Blixt MD, Wojciech Cebula MD, PhD, Lars Kristian Munck MD, DMSc., Anne-Marie Kanstrup Fiehn MD, DMSc.

Supplementary Table S1

Histopathological criteria used in the study for each of the diagnostic categories.

**Colonic mucosa**	
Normal/nonspecific	No or only minimal or focal chronic inflammation. Few neutrophils in lamina propria or in the surface or crypt epithelium
Chronic inflammation	Moderate or severe diffuse chronic inflammation in lamina propria without an increased number of intraepithelial lymphocytes (IELs) or a thickened subepithelial collagenous band
Acute inflammation	Acute inflammation in lamina propria with cryptitis, crypt abscess, erosion, or ulceration
LC	Chronic inflammation in lamina propria and > 20 IELs/100 epithelial cells
LCi	Chronic inflammation in lamina propria and >10 to ≤20 IELs/100 epithelial cells
CC	Chronic inflammation in lamina propria and a subepithelial collagenous band >10 µm. An increased number of IELs
CCi	Chronic inflammation in lamina propria and a subepithelial band >5 to ≤ 10 µm. An increased number of IELs
Neoplasia/cancer	Any kind of neoplasia
Others	I.e., granulomas or crypt irregularity
**Ileal mucosa**	
Normal/nonspecific	No or only minimal or focal chronic inflammation. Few neutrophils in lamina propria or in the surface or crypt epithelium. Hyperplasia or lymphoid aggregates. Minimal architectural distortion.
Chronic inflammation	Moderate or severe diffuse chronic inflammation in lamina propria without an increased number of IELs or a thickened subepithelial collagenous band
Acute inflammation	Acute inflammation in lamina propria with cryptitis, crypt abscess, erosion, or ulceration
MC/MCi	Chronic inflammation in lamina propria and >10 IELs/100 epithelial cells or a subepithelial collagenous >5 µm

Others	I.e., metaplasia or granulomas
No biopsies	No biopsies available from the ileal mucosa
